# Wild meat consumption in tropical forests spares a significant carbon footprint from the livestock production sector

**DOI:** 10.1038/s41598-021-98282-4

**Published:** 2021-10-07

**Authors:** André Valle Nunes, Carlos A. Peres, Pedro de Araujo Lima Constantino, Erich Fischer, Martin Reinhardt Nielsen

**Affiliations:** 1 Programa de Pós Graduação em Ecologia e Conservação, Instituto de Biociências, Universidade Federal de Mato Grosso do Sul, Campo Grande, Mato Grosso do Sul Brasil; 2 Instituto Nacional de Pesquisa do Pantanal, Programa de Capacitação Institucional, Museu Paraense Emílio Goeldi, Cuiabá, Mato Grosso Brasil; 3 RedeFauna – Rede de Pesquisa em Diversidade, Conservação e Uso da Fauna da Amazônia, Brasília, Brasil; 4 School of Environmental Sciences, University of East Anglia, Norwich, UK; 5 Instituto Juruá, Rua das Papoulas 97, Aleixo, Manaus, AM Brasil; 6 Department of Food and Resource Economics, University of Copenhagen, Frederiksberg C, Denmark

**Keywords:** Ecology, Biodiversity, Climate-change ecology, Conservation biology, Ecosystem services, Environmental economics

## Abstract

Whether sustainable or not, wild meat consumption is a reality for millions of tropical forest dwellers. Yet estimates of spared greenhouse gas (GHG) emissions from consuming wild meat, rather than protein from the livestock sector, have not been quantified. We show that a mean per capita wild meat consumption of 41.7 kg yr^−1^ for a population of ~ 150,000 residents at 49 Amazonian and Afrotropical forest sites can spare ~ 71 MtCO_2_-eq annually under a bovine beef substitution scenario, but only ~ 3 MtCO_2_-eq yr^−1^ if this demand is replaced by poultry. Wild meat offtake by these communities could generate US$3M or US$185K in carbon credit revenues under an optimistic scenario (full compliance with the Paris Agreement by 2030; based on a carbon price of US$50/tCO_2_-eq) and US$1M or US$77K under a conservative scenario (conservative carbon price of US$20.81/tCO_2_-eq), representing considerable incentives for forest conservation and potential revenues for local communities. However, the wild animal protein consumption of ~ 43% of all consumers in our sample was below the annual minimum per capita rate required to prevent human malnutrition. We argue that managing wild meat consumption can serve the interests of climate change mitigation efforts in REDD + accords through avoided GHG emissions from the livestock sector, but this requires wildlife management that can be defined as verifiably sustainable.

## Introduction

Hunting of wild meat (also called bushmeat hunting) remains controversial in conservation policy and science because its net effect is detrimental to game populations when unsustainable, but often culturally and nutritionally essential for the subsistence needs of local human populations^1–3^. Unsustainable hunting can have cascading effects that suppress the long-term carbon storage capacity of natural forests by depleting large-bodied bird and mammal species serving essential ecosystem functions, such as dispersal of large-seeded carbon-dense tree species^4,5^. Unsustainable hunting, therefore can lead to shifts in the species composition of tropical tree assemblages that ultimately reduce the forest carbon storage capacity^6–9^.

On the other hand, hunting can provide a sustainable source of protein and essential micronutrients if appropriately monitored and managed^10^. Forecasts predict widespread protein deficiency in a range of tropical countries, and case studies suggest increased risk of anaemia in children if wild meat is insufficient^11^ to the point where the prevalence of child growth stunting can be negatively related to game abundance^12^. Despite the perils of managing human-occupied tropical wildlands^13^, sustainable hunting can enable the persistence of healthy wildlife populations of even less resilient low-fecundity game species while simultaneously supporting local subsistence^14,15^. A systematic review of 628 game hunting assessments in tropical forest areas found that only one-third were unsustainable^16^. Furthermore, sustainable wild meat hunting in tropical forest landscapes requires the persistence of extensive intact forest areas^17,18^. The alternative of supplying meat demand through local beef production from ruminant livestock involves deforestation, with strikingly detrimental repercussions for both biodiversity conservation and carbon emissions^19^. Land-use conversion to croplands and cattle pastures is the principal driver of deforestation worldwide^20^. Cattle ranching is, for instance, directly responsible for 71% of all Latin American deforestation, and pasture expansion has been the single largest driver of deforestation across the region since the 1970s^21^. The expansion of the livestock production sector in tropical forest countries to supply the growing demand for red meat is a crucial driver of both biodiversity loss and greenhouse gas (GHG) emissions^22,23^. Beef and a few other red meats, for instance, supply 1% of the world's calories but account for 25% of all emissions occurring due to land-use change^24^. The livestock sector also disproportionally contributes to the environmental cost of agriculture through high resource misuse, including water, land, and soils^25^.

As the global human population is projected to grow from 7.8 billion people today to 9.2–9.9 billion by 2050, global food demand is expected to increase substantially^26^. Rapidly escalating food demand will further accelerate land-use change, driving biodiversity loss and releasing substantial amounts of additional carbon into the atmosphere^27^. Coupling conservation of biodiversity and natural ecosystem services such as carbon storage with global scale food production for a growing human population requiring ever more animal protein is, therefore, among the most critical challenges facing humanity today^28^.

Tropical forests fulfil an essential service by storing an estimated 460 billion tons of carbon, more than half the total atmospheric content^29^. However, only 20% of all remaining tropical forest areas, storing ~ 40% of the above-ground tropical forest carbon stock, can be defined as “intact”^30,31^. Across the vast Amazon basin, for instance, intact forest areas are concentrated mainly within indigenous territories and protected areas, which collectively store some 42 GtC^32^. Promoting sustainable wild meat utilization, rather than the current scenario of expanding domestic livestock production across the tropics at the expense of natural ecosystems, could generate a comparatively lower carbon footprint. However, the magnitude of GHG emissions spared from consuming wild meat, rather than protein from the livestock production sector, has not been quantified. Similarly, the potential carbon credits that could be generated through, for instance, REDD + payments is unknown.

REDD + (Reduced Emissions from Deforestation and forest Degradation) is a multilateral carbon credit trading mechanism enabling polluters in usually high-income countries to pay low-income countries for reducing deforestation and forest degradation^33^. This was proposed to reduce carbon emissions, conserve and enhance forest carbon stocks, and sustainably manage forests to the benefit of native biodiversity. However, although the United Nations Framework Convention on Climate Change (UNFCCC) highlights the importance of co-benefits in REDD + programs^34^, these have generally failed to recognize and incorporate subsistence hunting and the role of wild meat in forest governance^35,36^.

**Figure 1:**
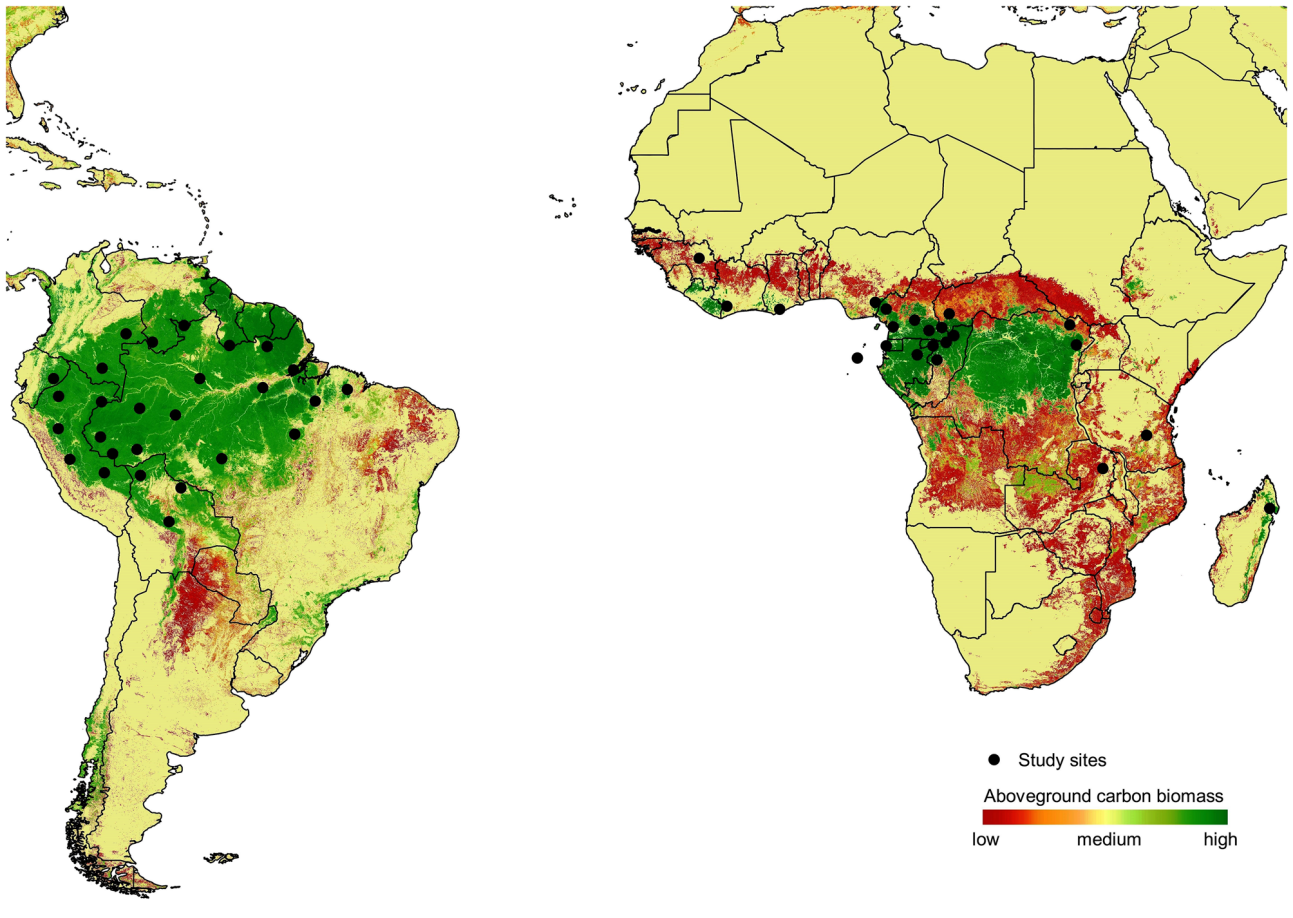
Geographic location of the 49 study sites compiled across seven South American and 14 African countries (including one site in Madagascar). The colour-coded background represents the distribution of above-ground forest biomass (range = 0–620 Mg ha^−1^)^84^. (Figure is created by QGIS 2.18.0, http://qgis.osgeo.org).

Here, we assess the carbon footprint forgone through wild meat consumption by human populations in 49 tropical forest study sites in 22 Afrotropical and 27 Neotropical countries (see Fig. 1). We estimate the number of carbon equivalents (CO_2_-eq) spared through wild meat consumption compared to the alternative of consuming bovine beef and poultry—the domestic animal most likely to replace wild terrestrial game consumption across the pan-tropics under future scenarios^37,38^. We further estimate the carbon credit value of emissions forgone using two scenarios: (A) the price necessary to provide incentives to reach the objectives of the Paris Agreement by 2030, and (B) a conservative carbon price. We find that wild meat consumption is associated with substantially reduced carbon emissions from the livestock production sector. Arguing that the sale of carbon credits based on carbon emissions forgone from livestock production can generate incentives to enhance tropical forest resource monitoring and management, we suggest that verifiably sustainable subsistence consumption of wild meat should be incorporated into future REDD + compensation schemes and climate change mitigation efforts.

## Results

### Wild meat consumption profiles

**Figure 2:**
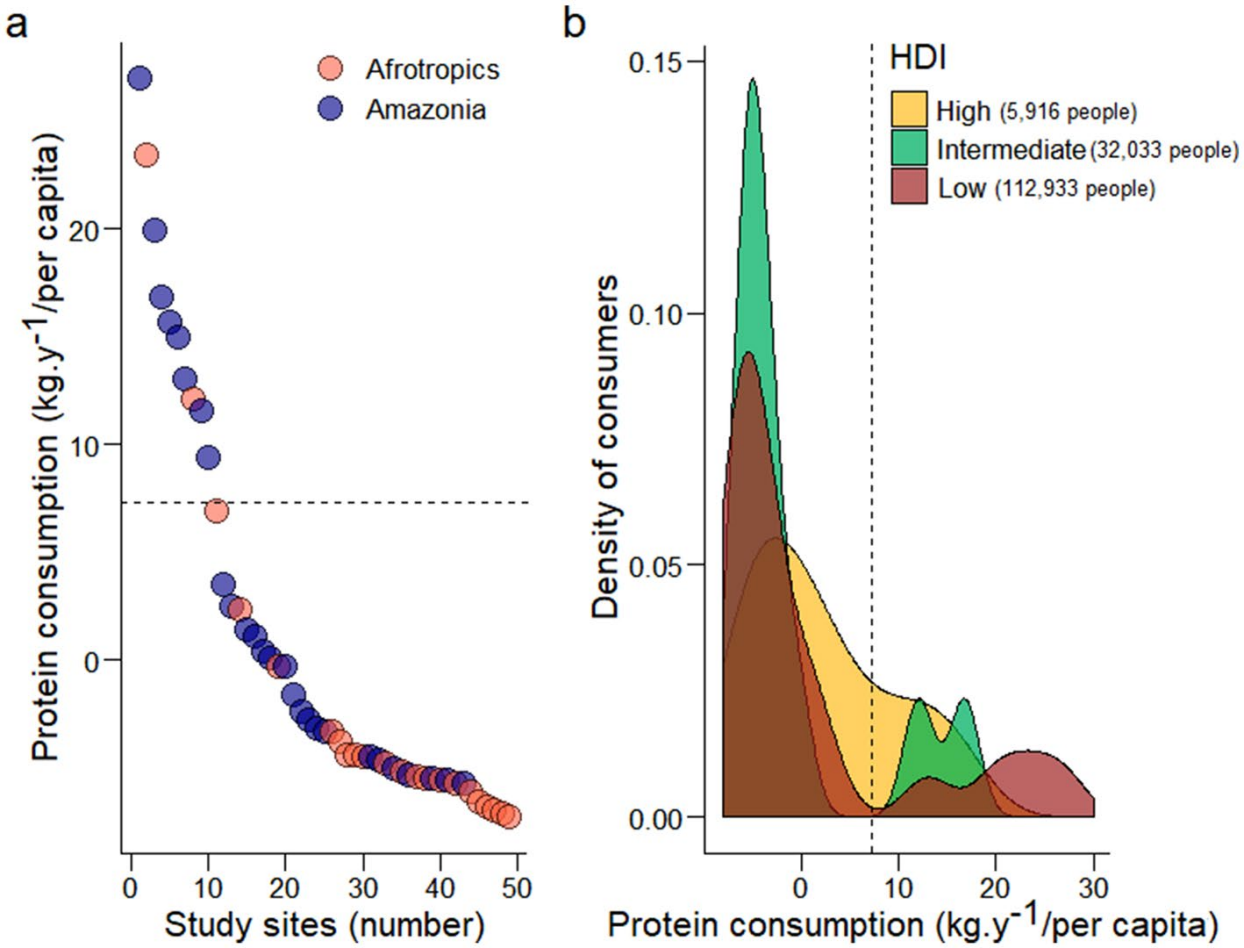
Distribution of mean annual per capita wild meat protein consumption rate in Amazonian and Afrotropical forest sites, ordered from the highest to the lowest consumption levels (**a**); and density distribution of consumers in relation to the Human Development Index (HDI) assigned to the smallest available subnational political unit in which study sites were located (**b**). Black dashed line represents the minimum annual per capita protein consumption of ~ 7.3 kg per person per year as recommended by FAO (2014) to prevent human malnutrition and/or under-nourishment.

A total of 250 terrestrial taxa were harvested across all 49 study sites, including 27 species in the Amazonian and 22 in the Afrotropical region. Mammals and birds were the most prevalent vertebrate classes in the harvest profiles, accounting for 64% and 27% of the numerical harvest across all sites, respectively. The total harvested biomass recorded by all studies represents 867,228 tons of undressed carcasses (mean ± SD = 17,698 ± 33,092 tons per site). This harvest corresponds to a biomass of 132,806 tons of animal protein (mean ± SD = 2,710 ± 5,671 kg per site), contributing to the nutritional health of the 150,882 people inhabiting the 49 sites. Mean (± SD) per capita consumption in the sample was 41.7 ± 48.1 kg person^−1^ yr^−1^ of wild meat and 8.3 ± 9.6 kg person^−1^ yr^−1^ of protein. The aggregate biomass of undressed carcasses amounted to 345,147 tons (mean ± SD = 12,782 ± 29,316 tons per site) consumed by 8,703 people in Amazonia, and 522,080 tons (mean ± SD = 23,730 ± 36,307 kg per site) consumed by 142,179 people in the Afrotropical sites. Overall, the mean per capita annual amount of wild meat protein consumed in Amazonia (11.0 kg ± 10.4 kg person^−1^ yr^−1^) was over two-fold higher than in Afrotropical forests (5.1 ± 7.3 kg person^−1^ yr^−1^; Fig. 2a).

Approximately 95% of all consumers in our sample were below the protein threshold recommended by the FAO. Most of these consumers were residents (63%) at the study sites (subnational units; country states/departments) which were burdened by low Human Development Index (HDI) scores (Fig. 2b).

### Avoided carbon footprint through wild meat consumption

**Figure 3:**
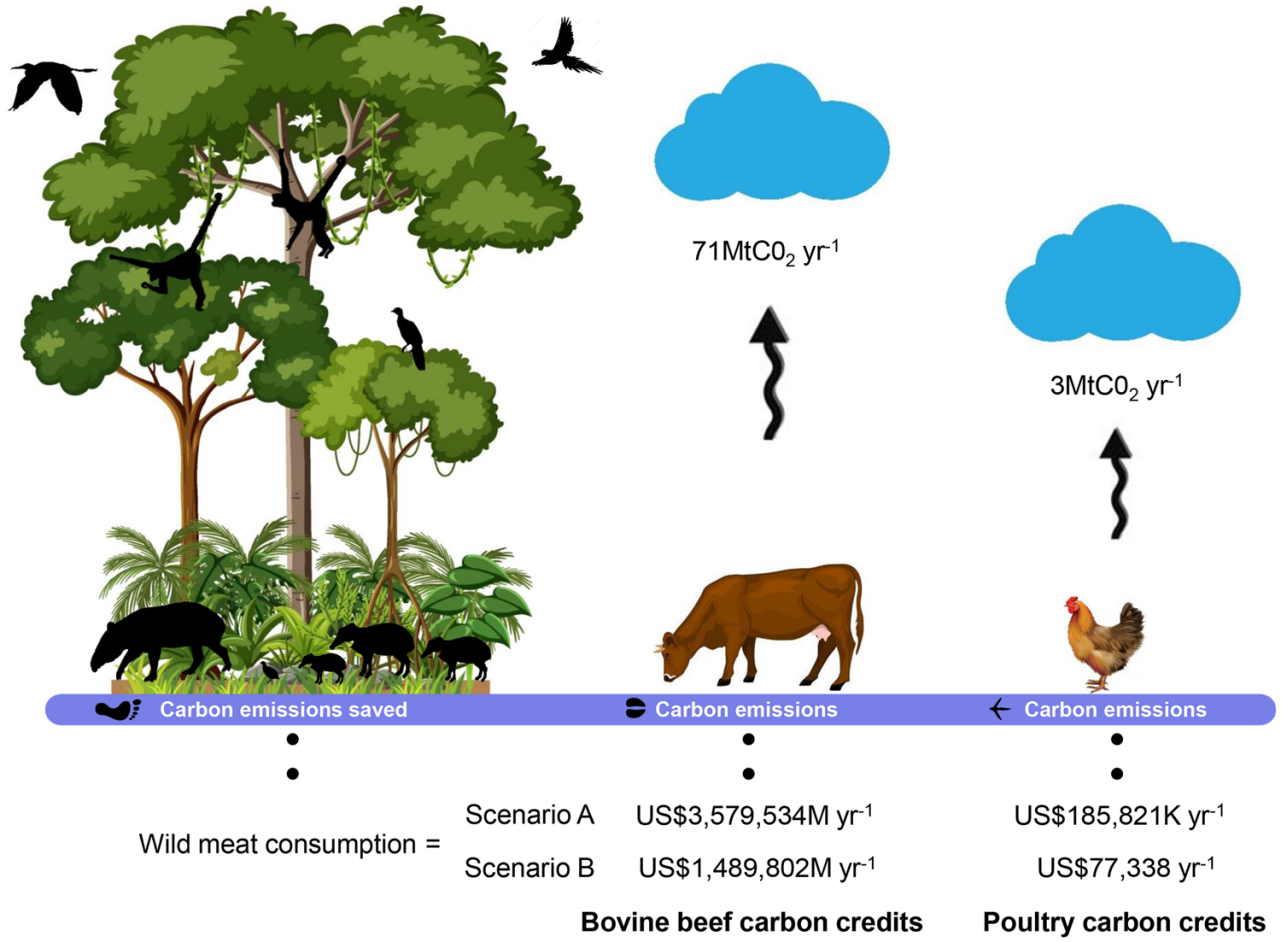
Potential value of carbon credits generated from wild meat consumption by the rural population across the 49 study sites in Amazonian and Afrotropical forests considered in this study, and the corresponding spared carbon footprint in terms of annual GHG emissions (tCO_2_-eq yr^−1^). Carbon prices are based on a value of US$50 per tCO_2_-eq under the optimistic Scenario A; and US$20.81 per tCO_2_-eq under the conservative Scenario B.

Replacing the estimated wild meat consumed by residents across all study sites by an equivalent biomass of undressed domestic animal meat would produce additional emissions of ~ 71 MtCO_2_-eq yr^−1^ given a bovine beef substitution scenario. These emissions would, however, be an order of magnitude lower at ~ 3 MtCO_2_-eq yr^−1^ should wild meat be replaced with poultry (Fig. 3). The avoided GHG emissions due to wild meat consumption was on average 1 MtCO_2_-eq yr^−1^ per site (± 3 MtCO_2_-eq) if replaced by bovine beef, but only 0.076 MtCO_2_-eq yr^−1^ per site (± 0.173 MtCO_2_-eq) if replaced by poultry. Using the 95% lower and upper confidence limits of these estimates, we calculated a 23–52 fold change in MtCO_2_-eq yr^−1^ and 0.124–twofold change in MtCO_2_-eq yr^−1^ for the bovine beef and poultry scenario, respectively. Breaking down avoided emissions to the level of individual consumers, the additional carbon footprint from the livestock sector would increase by 474 kgCO_2_-eq yr^−1^ per capita if local residents shifted their dietary intake to bovine beef. However, these emissions would be only 8.76 kgCO_2_-eq yr^−1^ per capita under a poultry substitution scenario.

### Future market-based transactions in carbon credits

Using the most optimistic Scenario A (US$50 per tCO_2_-eq), aiming to provide incentives to reach the objectives of the Paris Agreement by 2030, the income accrued from selling carbon credits derived from avoided GHG emissions—if the entire population across the study localities continues with the current consumption of an equivalent amount of wild meat rather than bovine beef—could potentially generate US$3,579,534 yr^−1^ across all sites. Site-level carbon credit values ranged from a minimum of US$1278 yr^−1^ to a maximum of US$1,062,091 yr^−1^. The alternative scenario of replacing wild meat with poultry would generate much lower carbon credits, equivalent to US$185,821 yr^−1^ across all sites, ranging from US$66 yr^−1^ to US$55,135 yr^−1^ per site. Our more conservative carbon credit price estimates (Scenario B; US$20.81 per tCO_2_-eq) could potentially generate US$1,489,802 yr^−1^, ranging from a minimum of US$532 yr^−1^ to a maximum of US$44,402 yr^−1^ for bovine beef. For poultry, these values would amount to only US$77,338 yr^−1^ across all sites, ranging from US$27 yr^−1^ to US$22,947 yr^−1^ per site (Fig. 3).

## Discussion

Our results highlight that replacing wild meat consumption by forest dwellers across the tropics with domesticated animal protein sources would massively increase the global human carbon footprint through additional emissions from the livestock sector. These estimated emissions forgone are conservative because they do not consider net carbon budgets beyond the farm gate. On the other hand, carbon emissions from manufacturing essential equipment for hunting activities (e.g. ammunition, shotgun, small motor vehicles) are not considered here. Moreover, protein replacements assuming purchases of domesticated meat have already been shown to be financially unfeasible for consumers in both Amazonian^3^ and Afrotropical forests^39^, who typically represent rural poor extractivists. Beyond the carbon footprint and nutritional implications, replacing wild meat as the leading source of protein would profoundly affect local economies and social customs through the symbolic value of wild meat in traditional cultures^40^. Our results further strongly suggest that managing forests for their carbon storage function, the primary objective of REDD +^34^, needs considerable rethinking. Quantifying and monitoring standing above-ground carbon stocks in the phytomass alone fails to consider the complex trophic interactions and dependence on wild game for food and income by millions of people outside the wage-labour sector of regional economies^36^.

Average per capita consumption was 41.7 kg of wild meat per year, corresponding to an average daily intake of 23 g of protein per person^−1^, representing 41% and 46% of the recommended per capita daily amount of protein required for adult men and women, respectively^41^. In only 37% of all sites, the local population consumed wild meat protein that either matched or exceeded the minimum protein consumption threshold recommended by FAO (7.3 kg person^−1^ yr^−1^). Information on other animal and plant protein sources contributing to human diets—including beef, poultry, fish and legumes—is unavailable. However, 37% of the study sites in mainland Africa and Madagascar and 26% in the Amazon failed to provide this minimum recommended amount of protein intake from wild meat alone (see Fig. 2a), amounting to 63% of all sites across all 49 sites. Geographic differences in wild meat protein consumption do not imply that wild meat intake in one tropical forest realm is less critical than in the other. On the other hand, the proportion of malnourished people is high in our dataset because we cannot reliably capture the variation in consumption between different age groups of local populations. While per capita consumption of wild game protein in Amazonia was high compared to Afrotropical sites, consumption levels in both regions were, for instance, higher than the annual per capita poultry consumption in both West Africa (where most sites were located) and South America, at an estimated 1.01 kg and 4.44 kg protein person^−1^ yr^−1^, respectively^42^. On a per-capita basis, wild meat consumption also exceeded bovine meat protein intake at an estimated 1.7 and 8.05 kg protein yr^−1^ in Central Africa and Amazonia, respectively^42^. Hence, although wild meat consumption alone may not ensure that protein intake match the rates recommended by FAO, wild meat remains critical in averting food insecurity and malnutrition in tropical forest regions while exerting a negligible or zero carbon-footprint beyond boat fuel, ammunition, and firearm production (but see ref. 36 for other more intangible potential implications).

Current consumption of wild meat represents spared emissions amounting to 4% of the MtCO_2_-eq emitted by beef production in the global bovine livestock sector serving a world population of 6 billion in 2005 (2836.8 MtCO_2_-eq emitted by the global scale beef production)^43^. This is equivalent to 0.05% of the 2018 global food supply chain, which resulted in ~ 13.7 billion metric tons of CO_2_-eq, or 26% of all anthropogenic GHG emissions^44^. To put this in perspective, this amounts to 751 Boeing-747 London—New York roundtrip flights, according to the International Civil Aviation Organization^45^. On the other hand, if we consider additional emissions associated with land-use change (LUC)—of 188 and 14 kg CO_2_-eq per kilogram of fresh bovine meat and poultry, respectively^46^—the spared carbon footprint from wild meat consumption would be 22% (320 MtCO_2_-eq yr^−1^) and 13% (22 MtCO_2_-eq yr^−1^) higher compared to our optimistic substitution scenario for bovine beef and poultry, respectively. These examples clearly show that maintaining healthy tropical forest game populations that can ensure sustainable wild meat consumption is an important piece of the climate change mitigation puzzle in developing countries.

Generating carbon credit revenues by including avoided GHG emissions from consuming wild meat in REDD + schemes could substantially contribute to local incentives for forest conservation and sustainable wildlife management through either direct payments for ecosystem services or other subsidies. To put these potential values into perspective, carbon credits generated considering the bovine beef replacement, for all 49 study sites, were equivalent to 3% and 1.23% (under Scenarios A and B, respectively) of the cost of the world’s largest tropical forest protection initiative—the Amazonian Protected Areas Program (ARPA)—which targets the Brazilian Amazon at an annual cost of US$121 million^47^. Saleable carbon credits generated under the more modest poultry replacement (Scenario A) alone would be ~ 12-fold greater than the financial costs of the monitoring, management, and dissemination components of this program^47^. At the scale of our 49 study sites, sales of carbon credits derived from wild meat consumption , assuming the high (optimistic) and low (conservative) carbon credit price in the bovine beef replacement scenario, represents 3% and 1.19%, respectively, of the Critical Ecosystem Partnership Fund budget, which supports conservation efforts across 19 tropical biodiversity hotspots in low-income countries (at US$125 million/2001–2003)^48^. To illustrate further, carbon credit sales at 49 sites, based on the bovine beef optimistic and conservative scenarios could annually fund 8% and 3% of households supported by the world’s largest Payment for Environmental Services (PES) program, the *Bolsa Verde*, which serves ~ 21,000 forest dwelling households (at US$44 million/2014)^49^.

Price uncertainty in carbon markets can be a disadvantage. This is defined not only in terms of short-term price volatility, but also includes the unpredictable behaviour of internal carbon prices used by enterprises, ranging from less than US$1/tCO_2_-eq to US$906/tCO_2_-eq^50^. If we apply this wide variation, our estimates would range from US$71 million to US$68 billion for bovine beef, and US$3 million to US$3 billion for poultry. Assuming the highest price bracket (US$906/tCO2-eq), the wild meat carbon footprint from bovine beef in Amazonian and Afrotropical forests would generate financial revenues 272% higher than the NASA (National Aeronautics and Space Administration) proposed budget for the 2021 fiscal year of US$25 billion^51^. This wide discrepancy underlines the degree to which we have been conservative in this analysis.

These examples highlight the considerable potential of carbon credit transactions to incentivize the de facto implementation of sustainable hunting initiatives in tropical forest regions. Throughout this process, such schemes would further release local beneficiaries from either purchasing or producing carbon-intensive domestic animal protein, such as bovine cattle and poultry. If channelled through appropriate institutional frameworks of PES and REDD + initiatives, these revenues could contribute to climate change mitigation efforts, as sustainable wild meat offtake is inextricably linked to large tracts of relatively intact tropical forest^52^. These initiatives could further promote the legal harvesting of sustainably managed game populations, thereby contributing to the food and nutritional security of rural populations at these localities, who experience a high prevalence of poverty and malnutrition. Taking this scenario into account, resource management policy should support local stakeholders in adopting both qualitative (e.g. which species can be harvested) and quantitative (e.g. sex, age and season harvest quotas) limits for the sustainable use of game vertebrate species based on forest area and its underlying secondary productivity. This should facilitate local capacity-building in straightforward game management regulation and local residents who could reinvest wild meat carbon benefits into positive welfare and education programs. Local authorities and villagers could additionally establish forest restoration plans that can further boost wild meat productivity, food security, and carbon credit benefits.

As the window for effectively curbing the magnitude of climate change is rapidly closing, concerted strategic planning of climate change mitigation efforts, biodiversity conservation and human well-being, including investments in low-carbon food production, is more critical than ever. No single solution can limit climate change^53^, but Community-Based Conservation Management (CBCM) may offer an essential piece of the puzzle. In Ethiopia, pastoral communities empowered to manage natural resources in the face of future climate change have been successful^54^. Similar successes of ecosystem-based approaches to climate change adaptation have been reported globally, including Togo and Sri Lanka^55,56^. However, little is known about the potential of CBCM to manage carbon stocks and ensure sustainable wild meat consumption in tropical forests.

The revenue potential of carbon credits from sustainable wild meat consumption can also support experimental CBCM tropical forest projects. Although CBCM should ideally be self-funded, community projects will often fail to get off the ground without considerable financial start-up support^57^. Carbon credit revenues proposed here could support the development and implementation of locally co-designed community-based monitoring schemes and enforcement of sustainable hunting regulations^15^. However, information is not available to enable us to confirm whether hunting is sustainable at all sites examined here. Additionally, little is known about what level of large-bodied frugivore populations declines may have occurred, thereby potentially inducing negative changes in forest carbon stocks^5^. Thus, maintaining populations of game species that can provide essential ecosystem services, such as effective seed dispersal, is essential to ensure functionally intact tropical forests^4,9^ that continue to generate co-benefits, including food security and climate change mitigation, thereby contributing to SDG 12.

Clearly, implementing a wild meat carbon credit scheme would not be unproblematic. If currently high capture rates cannot be sustained, hunters would have to reduce their offtake. Persuading hunters to incur such short-term opportunity costs, even just temporarily, would be difficult unless they can be compensated for any lost food or income^58^. In this case, part of the carbon credit revenue could be reserved for PES programs (e.g. *Bolsa Verde*) to offset these losses until wildlife populations recover. The current COVID-19 pandemic has generated a lively debate and proposals to ban wildlife trade and consumption altogether^59^. Interventions in response to COVID-19 and other zoonoses are required to minimize public health risks from disease transmission. However, instead of a blanket ban on subsistence hunting and wild meat trade, thereby eliminating an incentive for sustainable resource use [e.g. see Dickman et al. (2020)^60^ for a discussion concerning trophy hunting], hunters could be trained to monitor animal health as well as game populations^61^. The implementation of such programs could be a critical component in developing hygiene standards and routines for meat market surveillance in many countries, particularly in Africa, thereby contributing to SDG 3. Note, however, that other reforms of the wild meat sector are urgently needed in tropical countries and should be pursued concomitantly^62^.

## Conclusions

Using data from a large sample of studies on wild meat consumption in Amazonian and Afrotropical forests, we estimated considerable spared GHG emissions compared to plausible meat substitution scenarios involving protein replacements by bovine beef and poultry from the livestock sector. We then estimated potential revenues from the sale of associated carbon credits from consuming wild meat and discuss how this could generate financial incentives for forest conservation and sustainable wildlife management through PES and REDD + projects. Our results clearly illustrate the potential value and importance of considering sustainable game hunting within the REDD + political process at both national and international scales^37^. This challenge should be confronted in collaboration with local communities through community-based wildlife management projects to safeguard relatively intact forests, carbon storage, and long-term hunting yields. Enabling resource co-management by marginalized tropical forest communities will require transparency and devolution of tangible benefits from carbon credit revenues. Sustainable hunting can bring about considerable collective co-benefits in many tropical countries to local users in terms of increased food security and well-being, in addition to intangible benefits such as self-esteem, social engagement, and responsible resource stewardship that can spill over into future generations. Hence, carbon credits generated through sustainable wild meat offtake from natural forests can serve the often irreconcilable interests of wildlife conservation, local food security, forest governance, and international climate change mitigation efforts but will require verifiably sustainable wildlife management.

## Methods

### Study region

We compiled data from 49 studies conducted between 1973 and 2019 across 7 and 14 countries in Amazonian and Afrotropical forests, respectively (Fig. 1). These studies covered a combined area within these two tropical forest realms of ~ 6.8 million km^2^ within a mosaic of 29 ecoregions and 32 watersheds (Supplementary Table S1). Temperatures in the Amazon range from 25.8 to 27.9 °C. Annual precipitation at sites at the mouth of the Amazon River exceeds 3000 mm yr^−1^, but rainfall decreases from equatorial regions towards the tropics and the northwestern (1500 mm/yr) and in the inner Andean valleys (1000 mm/yr)^63^. Temperatures in Central African sites are less variable, ranging from 26 to 24 °C^64^, whereas precipitation ranges from 1000 to 1750 mm yr^−1^. Wetter areas are observed in equatorial regions where mean rainfall is as high as 2000 mm yr^−1^, including coastal regions of Cameroon, where the highest rainfall across the entire African continent has been recorded (> 2100 mm/yr^−1^)^65^. In East and West Africa, temperatures range between 24–20 °C and 25–28 °C, whereas annual rainfall ranges between 600–2000 mm and 25–2200 mm, respectively^66^.

The ~ 6.3 million km^2^ Amazon basin is arguably Earth’s most megadiverse region, with considerable variation in vegetation types and local vertebrate assemblages^67,68^. In Africa, the Congo Basin spans ~ 2.3 million km^2^, comprising 15% of all forests globally^69,70^. These forests harbour the highest alpha-diversity levels in mainland Africa and have been in the spotlight for several decades as one of the world's most threatened ecosystems^71^. Madagascar is one of the eight hottest biodiversity hotspots based on the level of plant and vertebrate endemics^72^. Rural populations in both regions have little access to infrastructure, goods, and public services and usually subsist on small-scale agriculture and forest resource extraction, including game vertebrates.

### Data acquisition and analysis

We searched for all studies on subsistence hunting in Amazonian and Afrotropical forests using the key words ‘hunting’, ‘bushmeat’, ‘wild meat’, ‘game species’, ‘consumption’, ‘Amazon’, and ‘Africa’ (and analogous terms in Portuguese, Spanish, and French) in peer-reviewed journals, technical reports, postgraduate dissertations, and secondary data through Google Scholar, Web of Science, and the Center for International Forestry Research (CIFOR) database (https://www.cifor.org/bushmeat/resources/bushmeat-database/). We included all spatially-explicit study localities that met the following criteria: studies that (1) reported on the entire assemblage of hunted species over a known period, (2) presented data on numerical offtakes (i.e. the number of prey items and/or biomass) or values of per capita consumption per unit time for each game species, (3) quantified the local consumer population size, and (4) spanned a minimum duration of 30 days of continuous sampling (mean ± SD sampling duration of studies = 323 ± 269 days, *N* = 49). From each study, we extracted information on study duration in days, geographic coordinates, the number of consumers, the total population size at the site where the study was carried out, and the species-specific numbers of individuals harvested.

Most studies failed to provide data on game species biomass harvested. We, therefore, converted the total number of animals harvested of each species at each site into a site-specific biomass estimate based on the mean adult body mass of each game species using information from the literature^73–75^. Undressed carcass yields were calculated by multiplying the hunted biomass by a factor of 0.6, representing the edible body mass, excluding viscera, skin, and skeletal material^76^. We then estimated the Daily Per-Capita Consumption (DPCC) of undressed game meat (kg × person^−1^ × day^−1^) at each study site based on the study duration and the number of consumers reported in each study, from Eq. (1) ^77^:

1 $$DPCC = \frac{{total\;undressed\;carcasses\;\left( {{\text{kg}}} \right)}}{{ consumers \left( n \right) \times study\;duration \left( {\text{d}} \right)}}$$

To estimate the annual undressed game biomass consumed by the entire population of each site, we multiplied the DPCC by 365 and the total consumer population size at each site. The daily and annual per-capita protein intake at each study site was estimated by assuming that protein represents 20% of the overall undressed carcass weight^78^. In addition, we assessed the extent to which consumption of wild meat contributes to preventing human protein deficiency. We used the minimum value of 7.3 kg, which represents the minimum annual per capita protein intake recommended by FAO^79^, and compared this to the observed mean annualized consumption at each site.

Scenarios for substituting wild meat by livestock consumption were defined based on a protein content of 25% and 12% for bovine beef and poultry meat, respectively^80,81^. To estimate the value of wild meat consumption in terms of omitted CO_2_-equivalent GHG emissions, we quantified the carbon footprint derived from each of these scenarios. We use bovine beef and poultry emission estimates, expressed as CO_2_-eq, following the Food and Agriculture Organization Corporate Statistical Database (FAOSTAT—http://www.fao.org/faostat/en/#data/EI), which estimates emissions in kg of CO_2_-eq per kg of domestic animal meat produced. This takes into account that GHG emissions used in the FAOSTAT are restricted to emissions generated at the farm level from production to the farm gate, thereby underestimating total emissions^42^. Additional emissions from upstream and downstream stages of the production, distribution, trade and consumption processes are not included in this study as we deliberately opted for the most conservative estimates. Furthermore, these additional emissions are highly variable depending on the local context and would therefore significantly increase uncertainty in our calculations. The carbon footprint of hunters at the sites we examined is exceedingly low because hunting forays require little equipment and are frequently conducted as incursions on foot, particularly if we consider that the alternative carbon footprint of domesticated meat production did not account for any emissions beyond the farm gate. Quantitative uncertainty analysis of the carbon footprint was calculated using 95% confidence intervals. Typically, this approach is used in GHG inventories emissions, according to the IPCC Guidelines applied to National Greenhouse Gas Inventories approaches^82^. We applied emission values for individual countries in 2017, the last year for which data are available in the database. We used mean GHG emission estimates of 54.13 CO_2_-eq kg (± 24.52 SD) per kg for bovine beef, and 2.81 CO_2_-eq kg (± 2.23 SD) per kg for poultry. We estimated the expected carbon footprint at each site under the scenario of wild meat consumption being replaced by domestic meat, i.e., the amount of annual CO_2_-eq kg^−1^ spared at each site through wild meat consumption, using Eq. (2):

2 $$Food\;carbon\;footprint = DPCC \times {\text{CO}}_{2} { - }eq\;{\text{kg}}\;domestic\;meat \times 365\;{\text{days}}$$

where DPCC is the daily per-capita consumption (Eq. 1), and CO_2_-eq kg of domestic livestock meat is the amount of CO_2_-eq emissions associated with farm-level production of either 1 kg of bovine beef or 1 kg of poultry. The value of spared carbon emissions (tCO_2_-eq) was estimated from Eq. (3):

3 $$Saleable\;carbon\;credits = food\;carbon\;footprint \times carbon\;credit\;value$$

where the carbon credit value is US$50/tCO_2_ (Scenario A), the carbon pricing considered necessary to provide appropriate incentives to reach the objectives of the Paris Agreement by 2030^83^. This value was selected because of the large variation in carbon transaction prices paid for REDD + credits in bilateral and multilateral schemes. However, as this price was suggested under the condition that a sufficiently ambitious climate policy environment is in place, it may overestimate the current carbon credit value. We therefore also calculated a scenario that takes into account more conservative carbon prices that, according to the IHS Markit Global Carbon Index, is comprised of prices from the California Compliance Allowance, RGGI, and European Allowance prices. This weighted global carbon price is equivalent to US$20.81/tCO_2_ (Scenario B). Furthermore, when estimating the carbon credit revenue potential, we assumed game exploitation to be functionally sustainable, thereby ignoring any detrimental effects that hunting-induced depletion may have on the long-term capacity of forest ecosystems to potentially retain or sequester carbon^36^.

## Supplementary Information

Supplementary Information.
