# Supplementary material for: Wild meat consumption in tropical forests spares a significant carbon footprint from the livestock production sector
Source: Sci Rep. 2021 Oct 7;11:19001. doi: 10.1038/s41598-021-98282-4 (PMC8497605; doi:10.1038/s41598-021-98282-4)
Supplement: Supplementary file 1 — Supplementary Information. [file 41598_2021_98282_MOESM1_ESM.docx]

Supplementary Material

**Wild meat consumption in tropical forests spares a significant carbon footprint from the livestock production sector**

Table S1. Game harvest studies obtained at 49 forest sites in different regions of the Amazonian and Afrotropical (see Fig. 1). The game offtake data were obtained to estimate wild-meat consumption and estimates of spared greenhouse gas (GHG) emissions.

| **Forest** | **Country** | **Region** | **Population (study site)** | **Wild meat**  **(consumption/kg)*** | **Source(s)** |
| --- | --- | --- | --- | --- | --- |
| Amazonia | Peru | Medio Javari (Nueva Esperanza) | 162 | 101.5 | Anonimous (2003) |
| Amazonia | Brazil | Dardanelos | 638 | 38.6 | Ayres and Ayres (1979) |
| Amazonia | Brazil | Gurupi and Turiaçu Rivers, Maranhão state | 99 | 10.2 | Balée (1985) |
| Amazonia | Brazil | Antimary State Forest | 150 | 22.6 | Calouro (1995) |
| Amazonia | Brazil | Katukina of the Biá River Indigenous land | 174 | 13.6 | Carneiro (2015) |
| Amazonia | Brazil | Paranatinga | 274 | 20.4 | Emidio-Silva (1998) |
| Amazonia | Peru | Alianza Cristina | 264 | 231.2 | Escobedo and Rios (2003) |
| Amazonia | Peru | San Rafael, Nuevo Lima, Alianza | 402 | 28.4 | Gavin (2007) |
| Amazonia | Peru | Comunal Yanesha Reserve | 877 | 37.0 | Gonzales (2003) |
| Amazonia | Colombia | Zancudo | 160 | 8.4 | Guarín (2010) |
| Amazonia | Venezuela | Chivacoa | 194 | 11.7 | Holmes et al*.* (1992) |
| Amazonia | Brazil | Apalai and Maxipurimo | 235 | 49.0 | Linke (2009) |
| Amazonia | Equador | Alto Corrientes | 58 | 9.0 | Chacon (2012) |
| Amazonia | Colombia | Aduche | 131 | 111.4 | Melo (1998) |
| Amazonia | Brazil | Riozinho da Liberdade Extractive Reserve | 138 | 120.4 | Nunes *et al.* (2019) |
| Amazonia | Peru | Tayakome | 332 | 20.1 | Ohl-Schasherer et al. (2007) |
| Amazonia | Brazil | Settlements along the Jarí River | 246 | 34.9 | Parry et al. (2009) |
| Amazonia | Brazil | A'Ukre | 133 | 43.2 | Peres and Nascimento (2006) |
| Amazonia | Brazil | Jaú National Park | 198 | 24.3 | Pezutti *et al.* (2004) |
| Amazonia | Brazil | Tapajós-Arapiuns Extractive Reserve | 468 | 13.7 | Reis et al. (2018) |
| Amazonia | Bolivia | Madre de Dios | 172 | 83.5 | Rumiz et al. (2001) |
| Amazonia | Guyana | Masakenari | 225 | 42.0 | Shaffer et al. (2017) |
| Amazonia | Brazil | Settlements along the Purus River | 492 | 54.2 | Tregidgo (2016) |
| Amazonia | Bolivia | Bella Vista | 423 | 7.9 | Winter (2002) |
| Amazonia | Bolivia | Chimoré | 73 | 115.2 | Stearman (1990) |
| Amazonia | Venezuela | Medio Caura | 1859 | 94.3 | Ferrer et al. (2012) |
| Amazonia | Peru | Native Community Bufeo, Pikiniki, Nuevo Belén | 126 | 135.7 | Pacheco and Amanzo (2003) |
| Afrotropics | Gabon | Ogooue-Ivindo Province | 130 | 8.5 | Lahm (1993) |
| Afrotropics | Equatorial Guinea | Rio Muni | 243 | 71.1 | Kümpel (2006) |
| Afrotropics | Central African Republic | Dzanga-Sangha Special Reserve | 2500 | 0.19 | Noss (1994) |
| Afrotropics | Cameroon | Banyang-Mbo Wildlife Sanctuary | 2019 | 12.3 | Willcox and Nambu (2007) |
| Afrotropics | Cameroon | Campo District | 60 | 96.8 | Dounias (1999) |
| Afrotropics | Cameroon | Dja Biosphere Reserve | 368 | 8.0 | Muchaal and Ngandjui (1999) |
| Afrotropics | Republic of Congo | Likouala region | 670 | 0.8 | Riddell (2013) |
| Afrotropics | Republic of Congo | Odzala National Park | 117 | 17.3 | Carpaneto and Germi (1989) |
| Afrotropics | Cameroon | Messok district, Department of Haut-Nyong, East region | 266 | 34.9 | Fa et al. (2016) |
| Afrotropics | Democratic Republic of Congo | Ituri Forest | 308 | 20.0 | Fa et al. (2016) |
| Afrotropics | Nigeria and Cameroon | Abragba | 1733 | 14.0 | Fa et al. (2016) |
| Afrotropics | Côte d'Ivoire | Taï National Park | 720 | 2.3 | Kouassi *et al*. (2019) |
| Afrotropics | Tanzania | Udzungwa Mountains | 11232 | 10.5 | Nielsen (2006) |
| Afrotropics | Ghana | Takorandi | 17513 | 3.6 | Cowlishaw et al. (2005) |
| Afrotropics | Democratic Republic of Congo | Azande | 3500 | 14.6 | Merode et al. (2004) |
| Afrotropics | Madagascar | Masoala Peninsula | 118 | 5.8 | Bogerson (2016) |
| Afrotropics | Cameroon | Lobeke National Park | 409 | 153.6 | Tieguhong and Zwolinski (2009) |
| Afrotropics | São Tome | São Tome | 819 | 1.3 | Carvalho et al. (2015) |
| Afrotropics | Cameroon | 50,51,52 km west of Moloundou, SE Cameroon | 88 | 14.6 | Hayashi (2008) |
| Afrotropics | Guine | Niger National Park | 8500 | 9.3 | Brugiere and Magassouba (2009) |
| Afrotropics | Republic of Congo | Sangha region | 400 | 9.1 | Mbete et al. (2010) |
| Afrotropics | Zambia | Luangwa National Park | 466 | 47.7 | Marks (1973) |

* Per capita annual wild meat consumption.

**References**

Anonimous. Undated. Expedición al río Yavarí del 30 de octubre al 08 de diciembre, 2003.

Ayres J. M. & Ayres C. Aspectos da caça no alto rio Aripuanã. *Acta Amaz.* **9**, 287-298 (1979).. <https://doi.org.10.1590/1809-43921979092287>

Balée, W. Ka'apor ritual hunting. *Hum. Ecol.* **13**, 485-510 (1985).

<https://doi.org/10.1007/BF01531156>

Bogerson, C. Optimizing conservation policy: the importance of seasonal variation in hunting and meat consumption on the Masoala Peninsula of Madagascar. *Oryx*. **50**, 405-418 (2016). <https://doi.org/10.1017/S0030605315000307>

Brugiere, D. Magassouba, B. Pattern and sustainability of the bushmeat trade in the Haut Niger National Park, Republic of Guinea. *Afr. J. Ecol.* **47**, 630-639 (2019). <https://doi.org/10.1111/j.1365-2028.2008.01013.x>

Calouro, A. M. Caça de subsistência: sustentabilidade e padrões de uso entre seringueiros ribeirinhos e não ribeirinhos do estado do acre. Thesis. Universidade de Brasília, (1995).

Carneiro, D. B. Como eu vivo, me sustento: Formas indígenas de usos de recursos naturais. Tese de Mestrado. UFOPA, (2015).

Carvalho, M., Rego, F., Palmeirim, J. M., & Fa, J. Wild meat consumption on São Tomé Island, West Africa: implications for conservation and local livelihoods. *Ecol. Soc.* **20**, 27 (2015). <https://doi.org/10.5751/ES-07831-200327>

Carpaneto, G. M. & Germi, F. The mammals in the zoological culture of the Mbuti Pygmies in north-eastern Zaire. *Hystrix.* **1**, 1-83 (1989). <https://10.4404/hystrix-1.1-3888>

Chacon, R. J. Conservation or resource maximization: Analysis of subsistence hunting among the Achuar (shiwiar) of Ecuador. In The ethics of anthropology and amerindian research(Eds. Chacon RJ, Mendoza RG). Springer. Pp. 311-360. (2012).

Dounias, E. Le câble pris au piège de la conservation. Technologie du piégeage et production cynégétique chez les Mvae du sud Cameroun forestier. In: Bahuchet S, Bley D, Pagezy H, Vernazza-licht N. editors. L’homme et la forêt tropicale. Travaux Société Ecologie Humaine, Paris; 1999. pp. 281–300 (1999).

Emidio-Silva, C. A caça de subsistência praticada pelos índios Parakanã (sudeste do Pará): Características e sustentabilidade. Master Thesis. UFPA, (1998).

Escobedo, A. J. G. & Rios, C. C. R. Uso de la fauna silvestre, peces y de otros productos forestales no maderables em las comunidades de las etnias quechua y achuar del rio Huasaga, Loreto-Peru. Thesis. Universidad Nacional de la Amazonia Peruana (2003).

Fa, J. E. *et al*. Differences between pygmy and non-pygmy hunting in congo basin forests. *PLoS ONE.* **11**,1–20 (2016).

<https://doi.org/10.1371/journal.pone.0161703>

Ferrer, A., Romero, V. & Lew, D. Consumo de fauna silvestre en el eje agrícola Guarataro, Reserva Forestal El Caura, Estado Bolívar, Venezuela. *Memoria de la Fundación La Salle de Ciencias Naturales,* **173-174**, 239-251 (2012).

Gavin, M. C. Foraging in the fallows: Hunting patterns across a successional continuum in the Peruvian Amazon. *Biol. Conserv*. **134**, 64-72 (2007). <https://doi.org/10.1019/j.biocon.2006.07.011>

Gonzales, J. A. Patrones generales de caza y pesca em comunidades nativas y asentamientos de colonos aledaños a la Reserva Comunal Yanesha, Pasco, Peru. In Manejo de fauna silvestre em Amazonia y Latinoamerica (Ed. Polanco-Ochoa R). CITES. Cartagena (2003).

Guarín, M. D. P. T. Evaluación de la sostenibilidad de la cacería de mamíferos en la comunidad de zancudo, reserva nacional natural puinawai, guainía-colombia.Tese. Universidad Nacional de Colombia. Thesis. Universidad Nacional de Colombia (2010).

Holmes, R. & Clark, K. Diet, acculturation and nutritional status in Venezuela's Amazon territory*. Ecolgy of Food Nutrition.* **27**, 163-187 (1992).

[https://doi.org/10.1080/03670244.1992.9991242](https://doi.org/10.1080/03670244.1992)

Kümpel, N. F. Incentives for sustainable hunting of bushmeat in Río Muni, Equatorial Guinea. PhD thesis, Institute of Zoology, Zoological. Society of London and Imperial College London, University of London; 2006.

Lahm, S. A. Ecology and economics of human/wildlife interaction in northeastern Gabon. Dissertation. New York University, New York, (1993).

Linke, I. H. V. V. Caracterização do uso da fauna cinegética em aldeias das etnias Wayana e Aparai na TI Parque Indígena do Tumucumaque. Tese. Universidad Federal do Pará (2009).

Mbete, P. *et al*. Evaluation des quantités de gibiers prélevées autour du Parc National d'Odzala-Kokoua et leurs impacts sur la dégradation de la biodiversité. *J. Anim. Plant. Sci*. **8**, 1061–1069 (2010).

Marks, S. A. Prey selection and annual harvest of game in a rural Zambian community. *Afr. J. Ecol*. **11**,113-128 (1973). <https://doi.org/10.1111/j.1365-2028.1973.tb00077.x>

Melo, N. J. H. Caracterizacion de los patrones de caceria em la comunidad de Aduche y el Assentamiento de Puerto Santander-Araracuara, médio Caquetá, Amazonia (1998).

Muchaal, P. K. & Ngandjui, G. Impact of village hunting on wildlife populations in the western Dja Reserve, Cameroon. *Conser. Biol*. **13**, 385–396 (1999). <https://doi.org/10.1046/j.1523-1739.1999.013002385.x>

Noss, A. J. Duikers, cables and nets: A cultural ecology of hunting in a Central African forest. Ph.D. thesis. University of Florida, Gainesville, FL, USA, (1994).

Nunes, A. V., Peres, C. A., Constantino, P. A. L., Santos, B. A., Fischer, E. Irreplaceable socioeconomic value of wild meat extraction to local food security in rural Amazonia. *Biol. Conserv*. **236**, 171-179 (2019).

<https://doi.org/10.1016/j.biocon.2019.05.010>

Ohl‐Schacherer, J., Shepard Jr, G.H., Kaplan, H., Peres, C.A., Levi, T. & Yu, D.W. (2007). The sustainability of subsistence hunting by Matsigenka native communities in Manu National Park, Peru. *Conser. Biol*. **21**, 1174-1185.

<https://doi.org/>[10.1111/j.1523-1739.2007.00759.x](https://www.researchgate.net/deref/http%3A%2F%2Fdx.doi.org%2F10.1111%2Fj.1523-1739.2007.00759.x)

Pacheco, V., Amanzo, J. Análisis de datos de cacería en las comunidades nativas de pikiniki y nuevo belén, río alto purús. In Alto Purus: Biodiversidad, conservacion y manejo (Eds. Pitman RL, Pitman N, Alvarez P). Center for Tropical Conservation. Pp. 217-225 (2003).

Parry, L., Barlow, J. & Peres, A. C. Allocation of hunting effort by Amazonian smallholders: implications for conserving wildlife in mixed-use landscapes. *Biol. Conserv*., **142**, 1777-1786 (2009).

<https://doi.org/10.1016/j.biocon.2009.03.018>

Peres, C. A. & Nascimento, H. S. Impact of game hunting by the Kayapó of south-eastern Amazonia: implications for wildlife conservation in tropical forest indigenous. *Biodivers. Conserv.* **15**, 2627-2653 (2006).

[https:/doi.org/10.1007/s10531-005-5406-9](https://doi.org/10.1007/s10531-005-5406-9)

Pezutti JCB, Rebelo GH, Silva DF, Lima JP, Ribeiro MC. A caça e a pesca no Parque Nacional do Jaú. In Janelas para a Biodiversidade no Parque Nacional do Jaú (Eds. Borges, 2004.).

Reis, Y. S., Valsecchi, J. & Queiroz, H. Caracterização do Uso da Fauna Silvestre para Subsistência em uma Unidade de Conservação no Oeste do Pará. *Biodivers. Bras.* **8**, 187-202 (2018).

<https://doi.org/10.37002/biobrasil.v%25vi%25i.796>

Riddell, M. Assessing the impacts of conservation and commercial forestry on livelihoods in northern Republic of Congo. *Conser. Soc*. **11**, 199-217 . (2013). <https://doi.org./10.4103/0972-4923.121002>

Rumiz, D. I. & Maglianesi, M. A. Hunting impacts associated with Brazil nut harvesting in the Bolivian Amazon. *Vida Silvestre Neotropical.* **10**, 19-2 (2001).

Shaffer, C. A., Milsten, M. S., Yakuma, C., Marauanru, E. & Suse, P. Sustainability and comanagement of subsistence hunting in an indigenous reserve in Guyana. *Conserv. Biol.* **31**, 1119–1131 (2017).

[https://doi.org./10.1111/cobi.12891](https://doi.org./10.1111/cobi.12891)

Stearman, A. M. (1990). The effects of settler incursion on fish and game resources of the Yuquí, a native Amazonian Society of Eastern Bolivia. *Hum. Organ.* **49**, 373-385. <https://doi.org/10.17730/humo.49.4.906547u862h5x566>

Tieguhong, J. C. & Zwolinski, J. Supplies of bushmeat for livelihoods in logging towns in the Congo Basin. *Journal of Horticulture and Forestry*, **5**, 065-080.

Tregidgo D. Fishing and hunting in the Amazon floodplain: linkages among biodiversity conservation, rural livelihoods and food security. PhD Lancaster University (2016).

Willcox, A. S. & Nambu, D. M. Wildlife hunting practices and bushmeat dynamics of the Banyangi and Mbo People of southwestern Cameroon. *Biol. Conserv*. **134**, 251-261 (2007). <https://10.1016/j.biocon.2006.08.016>

Winter, K. A. Subsitence use of terrestrial and aquatic animal resources in the tierra comunitaria de origen itonama of lowland Bolivia. PhD. University of Georgia (2002).
